# Academy of Stomatology

**Published:** 1901-05

**Authors:** 

**Affiliations:** Academy of Stomatology


### ACADEMY OF STOMATOLOGY.

A REGULAR meeting of the Academy of Stomatology was held at the rooms of the Academy, 1731 Chestnut Street, Philadelphia, on the evening of Tuesday, February 26, 1901, the President, Dr. J. T. Lippencott, in the chair.

A paper was read by Dr. James P. Nichol, of Philadelphia, entitled "The Construction of Artificial Crowns."

(For Dr. Nichol's paper, see page 302.)

### DISCUSSION.

*Dr. I. N. Broomell.*—I wish to emphasize one or two points which were brought out. The first one is that of using thin platinum as a backing. This is a method that I myself have employed for some time in almost the same way as the essayist. I have always been of the opinion that the force applied by the bending of the pins is the primary cause of checking. If the pins are riveted, a method which I believe is almost obsolete at the present time, the pins are drawn from the body of the porcelain, and if they are bent over with force, this pulling effect on the body of the porcelain is also exerted. Therefore it seems to me highly important to bend the pins very slightly.

I wish also to endorse the little apparatus which has been exhibited for the purpose of swaging up hastily, skilfully, and successfully not only cusps, but many other small parts that we may occasionally be called upon to use. The water-bag gives one an opportunity of swaging directly on the plaster model, facilitates work, and brings out a very finely swaged form.

*Dr. A. P. Fellows.*—I wish to make mention of a mode of investment which I find very convenient, rapid, and in many respects better than that to which we have been accustomed to use. I make use of it in backing teeth. I usually finish my backing before a piece is completed. I back my tooth with pure gold 34 gauge, cut off the tooth pins to within a short distance of the backing, with a sharp instrument nick the edges of the pins, and then with a piece of hard or soft rubber swage the backing against the tooth. This will make it conform to the facing, no matter how irregular it may be, and without buckling. The investment is made by soaking a piece of asbestos paper and dipping it in water until it becomes a pulpy mass. The porcelain facing is pressed into the asbestos, and its edges are brought a trifle above the porcelain. I can immediately apply the blow-pipe without waiting for it to dry, and in a few minutes have a backing that is hard to excel for closeness. Having flowed upon this pure gold,—a 22-carat gold,—the entire back of the crown is of the same metal that the balance of the crown is made of.

I attempted last evening to make a backing in the way I suggest. I chose my tooth, made my backing, invested it, and soldered the metal on all in ten minutes, and I had a perfect backing and without any checking. I think checking is due altogether to excess of borax, and not to contraction of metals. If a very slight amount of borax, as small as you can possibly use, be put on the gold wherever you wish to add gold or solder, it is quite sufficient when a high-carat gold is used. With a low-carat gold or base-metal it is possibly necessary to use a quantity of borax; but in cases where pure gold or 22-carat gold is used, a small amount only is necessary, and it should never be allowed to touch the porcelain. I do not believe it is possible to break the porcelain. I find that by cutting the pins very short indeed, when the piece is finished there is very little excess to dress off. When the pins are of full length the solder flows around them and fills the piece out too full. Cutting off saves a great deal of trouble, and the facing is perfectly firm. I believe it may even be cut level with the porcelain and still be strong enough.

In regard to seamless gold crowns and crowns which are to be had at the dental depots I wish to say just a word. I believe they are used largely, but I question very much, indeed, whether it is possible to get a good occlusion and a good fit around the root with such a crown. I do not see how it is possible to take a measurement of the root and choose a crown and fit that on correctly.

*Dr. W. L. Ellerbeck.*—I have a question to ask the essayist. I infer from his paper that he has subjected the facings of the different dental manufacturing companies to the furnace temperature, at their melting-point, without producing any checking or cracking. I would like to ask him if he ever ground the porcelains first and then subjected them to the furnace temperature to find out if that would have any effect on them?

*Dr. Nichol.*—I never subjected them after grinding, but I have seen facings that would break up in the fingers after being heated.

*Dr. Ellerbeck.*—Do you use dry or wet flux?

*Dr. Nichol.*—Both. In sweating up bands in crowns I like the wet flux, and I use flux called Sorosis. Of course, in uniting pieces of bridge-work, I think it is better to use the dry flux, because it is not absorbed by the investment.

*Dr. S. H. Guilford.*—It is a very encouraging sign that young men come forward and read papers before this society, as they have done for the past three or four months. I hope more will follow their example.

The paper was on a very practical subject, and has brought out several practical points. It is easy enough to describe the different kinds of crown- and bridge-work, but there are many things that we do not understand as fully as we should. One of these things is the causes of the cracking of porcelain. I have looked into that matter myself very carefully for a good many years, and have come to the conclusion that, in the great majority of cases, porcelains are cracked by the too liberal application of borax. Porcelain on crowns or bridge-work usually breaks in one or two directions. It will crack entirely through the tooth, either horizontally or vertically, in the direction of the pins, or else it will crack parallel to the backing.

The causes of these two kinds of breaks are different. When the tooth breaks in the line of the pins I think it is due to the unequal rates of expansion of the platinum and porcelain. It was shown how the coefficient of expansion of the platinum and porcelain are about the same. If we heat them up the same, I do not see how it is possible to break the tooth, but if the platinum pins are heated and the porcelain cools before, then we have a case of fracture. Where we have a fracture parallel to the backing, I think it is caused by the borax getting through the pin-holes beneath the backing. Many have a larger hole than the pin requires, or the holes are enlarged to rectify an error in punching. That affords a good opportunity for the borax to get in. If the hole is nearly the same as the pin, the borax is not so apt to get beneath the backing. When the porcelain has broken off and the remnant is removed, one can see on the under side of the backing a distinct layer of solder, with its bubbled surface proving that the borax had gotten in and the solder followed. When the borax and solder get in you have something that comes in contact with the tooth that is much harder. The result is that that portion of the facing next to it is expanded and broken away. The other part remains, because the pin holds it. I now overcome that by this plan. After the backing is made and fitted to the tooth, I remove it and then adapt a piece of No. 20 or 30 foil gold, thin, over the back of the facing by means of a cork. The backing is then put on and pressed down, and the excess of foil trimmed off. By this means the borax is prevented from getting in between this layer of gold-foil and the tooth itself. In regard to the backing, I have found that there is nothing softer or more adaptable than what we are pleased nowadays to call platinum-gold or crown-metal. This material is made of gold and platinum rolled together. I put the gold side against the porcelain when I desire the light effect of the gold.

I endorse what Dr. Fellows said with regard to soldering. Many have the impression that after a tooth is invested all the moisture must be gotten out of the investment before attempting to apply the blow-pipe; whereas it has been shown that a wet investment is the best kind with which to solder, for when heated the hot water heats the porcelain. By putting asbestos paper on a tooth you can readily solder it at once.

As to getting occlusion of a tooth, more particularly a hollow metal crown, I recall a clinic in which a gentleman fitted a band to a root and trimmed it off to clear the occlusion of the opposing tooth; then he contoured the band, and across the occlusal end he soldered a flat piece of gold, No. 32 gauge. He cut out the central portion, leaving a hole. He swaged a thin piece of thin pure gold to the form of a cusp (using any form tolerably near what he desired). This he tacked in positon with a bit of solder. He then placed the crown upon the root and allowed the patient to bite down upon it, then took it off and fitted the cusp more perfectly to the band and allowed the patient to bite again, by which means he secured a perfect occlusion. After that the cusps were reinforced with solder.

*Dr. Joseph Head.*—I was reminded of some experiences that would naturally come to one who has employed low- and high-fusing porcelains. I can hardly see that there should be any great danger of cracking during soldering, because there are hardly any teeth that I know of that are not fused at a point considerably higher than required in soldering, and carefully heating them again to a temperature considerably below the original fusing-point would in no way endanger their structure.

The point taken by Dr. Fellows that the investment-backing has no tendency to crack facings does not seem to be well taken. In my early experience with crown-work I attempted two or three times to carry the thin backing over the cutting edge of the tooth, and allowed the solder to flow over it, which made it of such rigidity that when the contraction of the solder took place it bit off the cutting edge of the crown as neatly as if I had used scissors. This fact would seem to indicate that, under certain conditions, the backing might be the cause of the checking of the porcelain. I think we have all noticed that where a tooth is surrounded on three sides by backing, and we are so unfortunate as to allow the solder to flow completely around that tooth and allow for the contraction of the metal, we are very likely to have serious checking.

What really brought me to my feet was the question of the liability of borax to crack porcelain. I think that is the real danger. I can give you my experience with it, and shall then give you a theory with which I am not wholly content, but it is the best that I am able to present, and will, therefore, be subject to your further investigation. I noticed that when joining the joints of gum teeth with a high-fusing body the result was thoroughly satisfactory, and the high-fusing body could even be flowed outside of the joint so as to blend with the surrounding porcelain, but if by any chance the baking was not quite satisfactory, and I did not want to use a third or fourth firing, for fear something would happen, my brother and I thought it would be well to use a low-fusing porcelain on that; and almost invariably where we used the low-fusing porcelain the high-fusing porcelain would crack. That has been my experience, and I think we had the same experience when we used the borax.

Now, I shall give you my theory. The high-fusing porcelain is more or less porous. The low-fusing porcelain, when used as a glaze, becomes very much like glass. When they are both heated, the high-fusing porcelain, being porous, expands to a certain extent, but the low-fusing porcelain expands first to a greater extent, being liquid, and then when the cooling occurs contracts much more than the body below, thus causing a tension which may produce the cracking.

*Dr. Fred. A. Peeso.*—My experience has been that the cracking of facings is caused almost invariably by using too much flux. In crown- or bridge-work, where there is a broken facing, you will find that where the flux has worked through around the pins and under the backing the under side of the backing is covered with solder. I think that is the experience of almost every one. There are times with the central incisors where gold backing next to the tooth may be preferred, but I think that in the majority of cases very thin platinum is better, as pure gold backing next to a tooth shows through the enamel.

*Dr. James Truman.*—The theory that flux and borax will crack a facing seems to me contrary to the experience that some of us had during the earlier days when metal plate-work was exclusively used and when we knew nothing of crown- and bridge-work or rubber-work. At that time it was always my habit, in backing up teeth, to invariably coat the inside of the backing with borax, for the purpose of carrying the solder directly around the pins. Occasionally a tooth would crack, but, as a rule, we never expected such a thing as checking a tooth. In my judgment the trouble lies probably as much as anything else with the investment and with the rapidity with which it is heated. We were taught to invest properly and to heat the investment up to a certain point before daring to place the flame upon the tooth. I do not see why a tooth should be cracked by heat if proper precautions are taken. I do not believe in the borax theory.

*Dr. Louis Jack.*—I might mention the method that was used in the days Dr. Truman spoke of. Then we made our investments of sand and plaster, but they were almost invariably gradually heated in vessels of charcoal to a red heat, and nearly to the melting-point of the solder, before the blow-pipe was applied. In that way we avoided the sudden application of heat and also the variable rates of expansion that would have taken place if the reducing flame were applied before the mass had become entirely hot. In many instances I have had a piece soldered in a charcoal receptacle entirely without the use of the reducing flame, and merely as the result of the gradual and full heating of the mass in the heated charcoal. In that way we would heat up block cases made up in three or four pieces. A block case, heated as now heated, would be invariably spoiled. It was necessary to heat or solder a block piece in the manner indicated almost to the soldering-point before the blow-pipe was put upon it.

*Dr. Guilford.*—I would like to ask Dr. Truman whether he coated the inside of the backing with borax, or whether he simply put it around the pin-holes?

*Dr. Truman.*—I coated the whole backing.

*Dr. Guilford.*—Did the solder flow in?

*Dr. Truman.*—Certainly.

*Dr. Guilford.*—I have never seen solder on the inside of the backing in a plate where the porcelain was cracked. I think in every crown or bridge the solder has been there. There is some reason for that.

*Dr. Truman.*—I never riveted my pins, always split them, and the solder will necessarily flow in between the pins and under the backing. It cannot be prevented.

*Dr. Jeffries.*—Dr. Truman's plan was mine exactly, and I did it for years that way, and always coated the inside of the backing with borax so as to allow the solder to draw through, and I very rarely had a cracked tooth. As to pin bending causing a strain upon the porcelain, it may be a good idea to flatten the pins with a file to enable them to bend easily.

*Dr. Eugene Pettit.*—I think ninety out of a hundred teeth are cracked by the backing running over the edge of the porcelain.

We find some irregularities of porcelain. The gold would be pushed down into them, and that is sufficient to cause cracking.

*Dr. R. H. Nones.*—It seems to me that there is a great difference between the terms "crack" and "edge." A tooth that is cracked is altogether different from the tooth that is edged, and the tooth split directly through is, in most instances, due to too rapid cooling, or too rapid heating of that tooth, and not to borax. I must confirm what Drs. Truman and Jeffries have both said. I cannot go back as far as they, but it was my practice and that of Dr. Faber, with whom I was associated, to not only punch the pin-holes large, but to countersink them inside and out, so that the solder would go in beneath that backing and clamp the pin in place. With borax we would actually flood. Yet to-day, with bridge-work, I use more care than I ever attempted with plate-work and run more chance of edging the tooth. I do not find the trouble to lie in breaking directly through of the tooth, but in this edging. I think both Dr. Head and Dr. Nichol found the right idea when they said that the tooth is encased in metal, and has not the chance to expand or to move away from contracting solder. In backing I use pure gold more than platinum, but if I desire a grayish color I use platinum; if I want a yellow tint I use the gold.

*Dr. William Trueman.*—The essayist's method of fitting the backing seems to be rather complicated. The same result would be obtained much more accurately by the use of cornmeal. It is done in a very few moments, and backing can be used as heavy as 26 gauge and a perfect fit made. I have seen on the outside of such a backing the stamp of the numbers which the makers have upon the teeth.

I prefer in crown-work an alloy of gold and platinum,—twenty-two parts of pure gold and two parts of platinum. It is quite pliable, quite as much so as pure gold or pure platinum. It is a strong metal and does not show so much in the mouth; it has a peculiar gray color, and can be soldered with pure gold with safety; it is dense and takes a good polish. I find it satisfactory for making seamless crowns and for making caps in the porcelain-faced crowns. The essayist mentioned that by the sweating system a joint does not show. That, however, is not the case. I find it does show. Take a plate that has been sweated, put it under the blow-pipe, and you will see the line of union. After it has been worn it shows almost as plainly as solder. In the old time, when we got out our own gold, in cutting the plate we could economize by having it joined, and in that case the joint always showed. Why, I do not know, but it always did. It has the advantage over soldering the joint that it does not open upon any subsequent soldering. The seamless crown, I think, is far superior to the one that is built up; it is very much easier to contour the block of plaster than the gold. We can get the contour, make it fit the root and also the place.

I have brought here a practical case and crown. It is very much like the Butler process. The root is cut down by the trephine, and then there is a draw-plate, by which a cap can be swaged up. Then the pin is soldered in the tooth and the tooth fitted. I have one here. It is for a practical case. These little caps all ready to fit can be made and kept on hand, and they fit accurately.

I was interested in the remarks made in reference to the old-fashioned way of soldering. My way was that as described by Dr. Truman. We finished the backing and spread it all over with borax, then took a camel's-hair brush and spread the tooth on the inside and put the backing in place. I can confirm what Dr. Truman said, that the accidents we have now were then comparatively rare. We placed the case in the furnace and heated it, and when it reached the critical stage, kept a watch on it. When the right time arrived the soldering was done as quickly as it would take time to tell it. Accidents were comparatively rare. I am disposed to think that teeth at the present time are not of the same composition, for they split when I let the borax get inside the backing.

*Dr. James Truman.*—I do not think the cause of cracking is in the porcelain. I have been soldering teeth all through my professional life, and have used a great variety of teeth manufactured under different formulæ. Another cause must be found for this checking.

*Dr. W. L. Ellerbeck.*—In relation to the matter of borax causing the cracking of porcelain facings, I feel in accord with Dr. Truman on that matter, and I can say that the borax, which we know of as sodium biborate, may unite chemically with the silicate of porcelain, causing a lower-fusing silicate. I cannot conceive how it causes cracking. If put upon the basis of a lower-fusing body, as suggested by Dr. Head, and the difference in contraction is the occasion for the cracking, I should say that the borax would crack and not the facing. It seems to me that if close adaptation could be secured, that would effectually prevent any borax from getting in.

*Dr. Head.*—Dr. Ellerbeck has made some interesting remarks about the borax uniting with porcelain and making a lower-fusing porcelain. That seems to me very interesting, but it has been the experience of porcelain workers that a very much lower-fusing porcelain flowed over a high-fusing porcelain tends to crack it. If the borax unites with the porcelain and forms a lower-fusing body, we would not have the borax to contend with, but the lower-fusing porcelain.

*Dr. James P. Nichol.*—I am still of the opinion that the greater majority of facings are broken up in the soldering. In view of the fact that I was to read a paper here to-night, as stated, I made a few experiments. I backed up some facings and invested them. I then put the investment on the furnace and heated it enough to lift the wax out in a piece. I then threw the whole flame of the blow-pipe on the backings and investment and checked two facings out of four. When I heated up the investment gradually, I did not have any checking of the facings. I did not use any borax at all in either case. In regard to backing up a tooth, there seems to be some difference of opinion in reference to the overlapping. I leave the backing a little bit low on the incisive edge. Answering Dr. Broomell in regard to splitting the pins and bending them down, I think a good many are broken by using the burnisher for bending down the pins. If you take the pin-roughening pliers and mash the pin, it is easy to bend it down without crowding it too hard against the facing. I may have conveyed the wrong idea in speaking about burnishing. We take an incisor tooth and let the backing stand out a little all around. Snip along the cutting edge, and if properly invested it will be held in position until soldered. I do not approve of the crown swaging methods, and would suggest that it would be a good point for younger men to try to manipulate metals rather than to swage them.

Otto E. Inglis,
*Editor Academy of Stomatology.*

